# Prescription patterns of traditional Chinese medications and potential consequences in patients with new-onset cardiac or vascular-related diseases: a nationwide cohort study

**DOI:** 10.1186/s12906-025-04945-4

**Published:** 2025-07-02

**Authors:** Sheng-Shing Lin, Hsin-Hui Tsai, Daniel Hsiang-Te Tsai, Chiu-Lin Tsai, Nanae Itokazu, Jaung-Geng Lin, Edward Chia-Cheng Lai, Hsiang-Wen Lin, Yu-Chang Hou

**Affiliations:** 1 Graduate Institute of Chinese Medicine, College of Chinese Medicine, China Medical University, Taichung, Taiwan; 2 Department of Chinese Medicine, China Medical University Hospital, Taichung, Taiwan; 3 Department of Pediatric Neurology, China Medical University Children’s Hospital, Taichung, Taiwan; 4 Department of Pharmacy, Tai-An Hospital, Taichung, Taiwan; 5 School of Pharmacy, Institute of Clinical Pharmacy and Pharmaceutical Sciences, College of Medicine, National Cheng Kung University, Tainan, Taiwan; 6 Population Health Data Center, National Cheng Kung University, Tainan, Taiwan; 7 Department of Pharmacy, China Medical University Hospital, Taichung, Taiwan; 8 Nihon Pharmaceutical University, Saitama, Japan; 9 School of Pharmacy and Graduate Institute, College of Pharmacy, China Medical University, Taichung, Taiwan; 10 Department of Pharmacy System, Outcomes and Policy, College of Pharmacy, University of Illinois at Chicago, Illinois, USA; 11 Department of Chinese Medicine, Taoyuan General Hospital, Ministry of Health and Welfare, Taoyuan, Taiwan; 12 Department of Bioscience Technology, Chung Yuan Christian University, Taoyuan, Taiwan

**Keywords:** Cardiac disease, Vascular disease, Thromboembolism, Chinese medication, Prescription patterns

## Abstract

**Background:**

The patterns of Chinese medicine prescriptions, corresponding diagnoses, co-morbidities, and Western medication (WM) use among patients with cardiac or vascular-related diseases are uncertain. This research aimed to examine the patterns of Chinese medications (CMs, specifically in terms of extract granules), corresponding diagnoses, co-morbidities, and the use of WMs within specified follow-up periods among patients with potential of recurrent cardiac or vascular-related diseases and relevant outcomes.

**Methods:**

We conducted a retrospective cohort study using Taiwan’s National Health Insurance Research Database. We enrolled patients with newly diagnosed cardiac or vascular-related diseases without cancer(s), transplantation, bleeding diagnoses, or catastrophic illness during the 2-year period prior to the corresponding diagnosis. Prior and non-prior CM users were matched based on their propensity scores. Finally, we compared the CM and WM patterns prescribed by physicians, and co-morbidities in the 6 months following the diagnosis and the secondary cardiac or vascular-related events in the 2 years following the diagnosis between the two groups using the standardized mean difference.

**Results:**

Of 191,025 patients with newly diagnosed cardiac or vascular-related diseases, 39,341 (20.60%) were prescribed CMs. Moreover, after propensity score matching, we identified 39,168 prior CM users and 39,168 non-prior CM users. Regardless of prior CM use, both groups had a relatively high rate of comorbidities; CM or specific WM use; and incidence of severe cardiovascular, cerebrovascular, or thromboembolic events (33.81% vs. 31.97%) and severe bleeding (18.32% vs. 16.57%). Only CM exposure within 6 months after the index date differed significantly between the groups (73.51% vs. 30.34%).

**Conclusion:**

We found that over 30% of patients with newly diagnosed cardiac or vascular disease initiated CM use, while 73.5% of prior CM users continued. This finding highlights the need for healthcare professionals to carefully assess the risk-to-benefit ratio of CM use alongside WMs for patients with cardiac or vascular-related diseases.

**Supplementary Information:**

The online version contains supplementary material available at 10.1186/s12906-025-04945-4.

## Background

Cardiac or vascular-related diseases, including cardiovascular disease (CVD), cerebrovascular disease (CeVD), and venous thromboembolism (VTE), pose a major public health burden throughout the world [1–3]. In Taiwan, individuals with newly diagnosed myocardial infarction, ischemic stroke, or intracerebral hemorrhage frequently experience recurrent events within the first year, resulting in a substantial economic burden, with an estimate cost for a nonfatal event of US$2,100–2,900 [4].

In this context, traditional Chinese medicine (TCM) remains a common treatment option in Taiwan and is largely covered by National Health Insurance (NHI). Patients might seek TCM services—including two common dosage forms of Chinese medicines (i.e., the extract granule format or a decoction of crude Chinese medication [CM] materials), acupuncture, and/or traumatology—to manage cardiac and vascular-related diseases.Unlike Western medications (WMs), which typically involve fixed-dose, single active ingredients targeting a specific condition (e.g., an antihypertensive agent), any type of TCM treatment focuses on Zheng (syndrome) differentiation and treats the patient holistically, viewing the body as an interconnected system, rather than physiological or psychological symptoms of diseases [5]. For example, acupuncture or CMs (many of which comprise several ingredients or represent combinations) are prescribed to treat a patient’s overall responses but not for the symptoms of the target disease(s) or target organ(s) [6]. Some studies have investigated the concurrent use of NHI-covered extract granules of CMs and WMs for cardiac or vascular-related conditions, but the findings have been inconsistent [7–9]. These discrepancies may be due to the limited sample sizes, heterogenous study populations, varying disease severities, and differences between WMs and CMs [10].

More than 60% of NHI-insured individuals have used TCM services, such as acupuncture or CMs, with 86% of TCM visits resulting in prescriptions of CMs [11]. Among patients newly diagnosed with stroke, 12% sought TCM services, and over half of them received both CMs and acupuncture/traumatology [12]. Similarly, 28% of patients with diabetes and intracerebral hemorrhage were prescribed NHI-covered CMs [13]. Given these diverse prescription patterns and the uncertain clinical outcomes associated with CM use, this nationwide population-based study aimed to examine the usage patterns of CMs (specifically focused on extract granule formats of CMs), associated diagnoses, and comorbidities, and the concurrent use of WMs over a defined follow-up period among patients with a tendency to encounter recurrent cardiac or vascular-related diseases and relevant events.

## Methods

### Data sources

This study was approved by the Institutional Review Board of the China Medical University Hospital (CMUH104-REC3-024). We used Taiwan’s National Health Insurance Research Database (NHIRD), the details of which have been described elsewhere [14, 15]. Briefly, the database is derived from Taiwan’s NHI program, which covers approximately 23 million individuals (nearly 100% of the population). The NHIRD provides de-identified longitudinal medical and prescription information for research purposes [14]. We analyzed a randomly selected sample of 2 million individuals enrolled in the NHI program and excluded those who were under 20 years of age (to focus only on adults) or had no medical claim records from the analysis. Because almost all of CMs are reimbursed by NHI, we believe that the NHIRD provides relatively complete records regarding the use of TCM, including CMs [12, 16, 17].

### Study subjects and variables

Fig. S1 presents the study design and the specific follow-up periods. We included patients with newly diagnosed cardiac or vascular-related diseases (including CVD, CeVD, and VTE) between 2005 and 2011. We identified these relevant diagnoses using the International Classification of Diseases, Ninth Revision, Clinical Modification codes (Table S1). We included incident cases of CVD, CeVD, and VTE to ensure that all patients had a similar disease status. We excluded patients with cancer(s), a history of transplantation, a catastrophic illness certificate (for severe and disabling diseases), or bleeding-related diagnoses. The index date for each patient was defined as the date of new-onset CVD, CeVD, or VTE diagnosis. Patients were classified as prior or non-prior CM users based on dispensing records of CMs within the 6 months preceding the index date. We categorized CMs into formulas (referred to as CM formulas or Fu fang) and single CMs (referred to as single CMs or Dan fang) and listed their pinyin and botanical names, classification, efficacy based on TCM theory—as documented in the official reports [18, 19]—and preparation details in Tables S2 and S3.

### Follow-up assessments and potential outcomes

The primary assessment was the top 20 most frequently prescribed CM formulas and single CMs in the 6 months prior to the index date. The secondary assessments were medication use (including relevant WMs and CMs) and co-morbidities in the 6 months following the index date, and secondary cardiac or vascular-related events (including all-cause mortality, severe CVD/CeVD/VTE events, revascularizations, and severe bleeding) during the 2 years following the index date. The mortality data provided by the NHIRD are comprehensive, as confirmed in a previous study [20]. Patients were followed from the index date until the occurrence of an outcome of interest, death, or the end of the study period (December 31, 2013), whichever came first.

### Covariates

We selected covariates based on a literature review [12] and expert opinions. We captured the baseline covariates within 2 years prior to the index date, including sex, age range, insured amount, residential area, insured unit, urban level, disease history (e.g., peripheral vascular disease, dementia, chronic obstructive pulmonary disease, connective tissue disease, ulcer disease, mild liver disease, diabetes, diabetes with end-organ damage, hemiplegia, moderate/severe renal disease, moderate/severe liver disease, acquired immunodeficiency syndrome), and medication history (e.g., anti-glycemic drugs, cardiovascular drugs [antihypertensives], antithrombotic drugs, non-steroidal anti-inflammatory drugs, histamine type-2 receptor antagonists (H2 blockers) or proton pump inhibitors, steroids, antidepressants).

### Statistical analysis

We used SAS, version 9.4 (SAS Institute, Cary, NC, USA) for data management and statistical analysis. We analyzed the demographic characteristics; the categories of CMs used before and after the diagnosis of new-onset CVD, CeVD, or VTE; and the frequencies of patients receiving CM formulas and single CM prior to the index date. We considered a standardized mean difference (SMD) of − 0.1 to 0.1 to be a practically irrelevant difference between the prior and non-prior CM users [21]. Furthermore, we performed propensity score matching using all covariates listed in Table 1, applying greedy nearest-neighbor matching without replacement and a caliper width of 0.1, to facilitate comparability between the groups.

**Table 1:** Comparisons of patients’ characteristics prior the index dates for those who were prior or prior non-CM users after being matched with propensity score among those patients being diagnosed with new-onset cerebrovascular- or cardiovascular-related diseases

Characteristics identified prior the index date	After propensity score matching		
	Prior CM users (*n* = 39,168)	Non-prior CM users (*n* = 39,168)	SMD
Female^a^	22,470 (57.37%)	22,711 (57.98%)	-0.01
Age range^a^			
< 40	9156 (23.38%)	9252 (23.62%)	-0.01
40–50	8046 (20.54%)	7872 (20.10%)	0.01
50–65	14,022 (35.80%)	13,852 (35.36%)	0.01
>=65	7944 (20.28%)	8192 (20.92%)	-0.02
Insured amount^a^			
≤ 17,280	17,494 (44.66%)	17,628 (45.01%)	-0.01
17,281–28,800	14,074 (35.93%)	14,167 (36.17%)	0
28,801–45,800	4749 (12.12%)	4595 (11.73%)	0.01
45,801–72,800	2117 (5.40%)	2045 (5.22%)	0.01
≥72,801	734 (1.87%)	733 (1.87%)	0
Residential area^a,b^			
North	17,075 (43.59%)	17,430 (44.50%)	-0.02
Central	9125 (23.30%)	8270 (21.11%)	0.05
South	11,961 (30.54%)	12,357 (31.55%)	-0.02
East	945 (2.41%)	1058 (2.70%)	-0.02
Insured unit^a,b^			
Government/school employees	4467 (11.40%)	3871 (9.88%)	0.05
Private enterprise employees	14,210 (36.28%)	14,061 (35.90%)	0.01
Member of occupational	8312 (21.22%)	8139 (20.78%)	0.01
Farmers/fishermen	6766 (17.27%)	7259 (18.53%)	-0.03
Low-income, household, others	296 (0.76%)	305 (0.78%)	0
Urban level^a,b^			
1	11,070 (28.26%)	11,218 (28.64%)	-0.01
2	11,245 (28.71%)	11,332 (28.93%)	0
3	6291 (16.06%)	6127 (15.64%)	0.01
4	5767 (14.72%)	5691 (14.53%)	0.01
5	620 (1.58%)	745 (1.90%)	-0.02
6	1493 (3.81%)	1524 (3.89%)	0
7	1383 (3.53%)	1289 (3.29%)	0.01
Disease history			
Acquired Immunodeficiency Syndrome	9 (0.02%)	6 (0.02%)	0
Chronic obstructive pulmonary disease^a^	5445 (13.90%)	5389 (13.76%)	0
Connective tissue disease^a^	1254 (3.20%)	1085 (2.77%)	0.03
Dementia^a^	310 (0.79%)	234 (0.60%)	0.02
Diabetes^a^	4677 (11.94%)	4523 (11.55%)	0.01
Diabetes with end organ damage	1900 (4.85%)	3119 (7.96%)	0.03
Hemiplegia^a^	757 (1.93%)	703 (1.79%)	0.01
Mild liver disease^a^	1076 (2.75%)	898 (2.29%)	0.03
Moderate/severe liver disease	30 (0.08%)	27 (0.07%)	0
Moderate/severe renal disease	454 (1.16%)	396 (1.01%)	0.01
Peripheral vascular disease^a^	637 (1.63%)	550 (1.40%)	0.02
Ulcer disease^a^	7394 (18.88%)	7458 (19.04%)	0
Medication history			
Antidepressants	968 (2.47%)	819 (2.09%)	0.03
Antiglycemic drugs	3209 (8.19%)	3119 (7.96%)	0.01
Antithrombotic drugs	36 (0.09%)	29 (0.07%)	0.01
Cardiovascular drugs (antihypertensives)	14,195 (36.24%)	14,180 (36.20%)	0
H2 blockers or proton pump inhibitors	7665 (19.57%)	7693 (19.64%)	0
Non-steroidal anti-inflammatory drugs	24,544 (62.66%)	24,789 (63.29%)	-0.01
Steroids	5923 (15.12%)	5839 (14.91%)	0.01

^a^ Variables included in calculation of propensity score; ^b^ A small number of individuals were not classified into the predefined categories

## Results

**Fig. 1:**
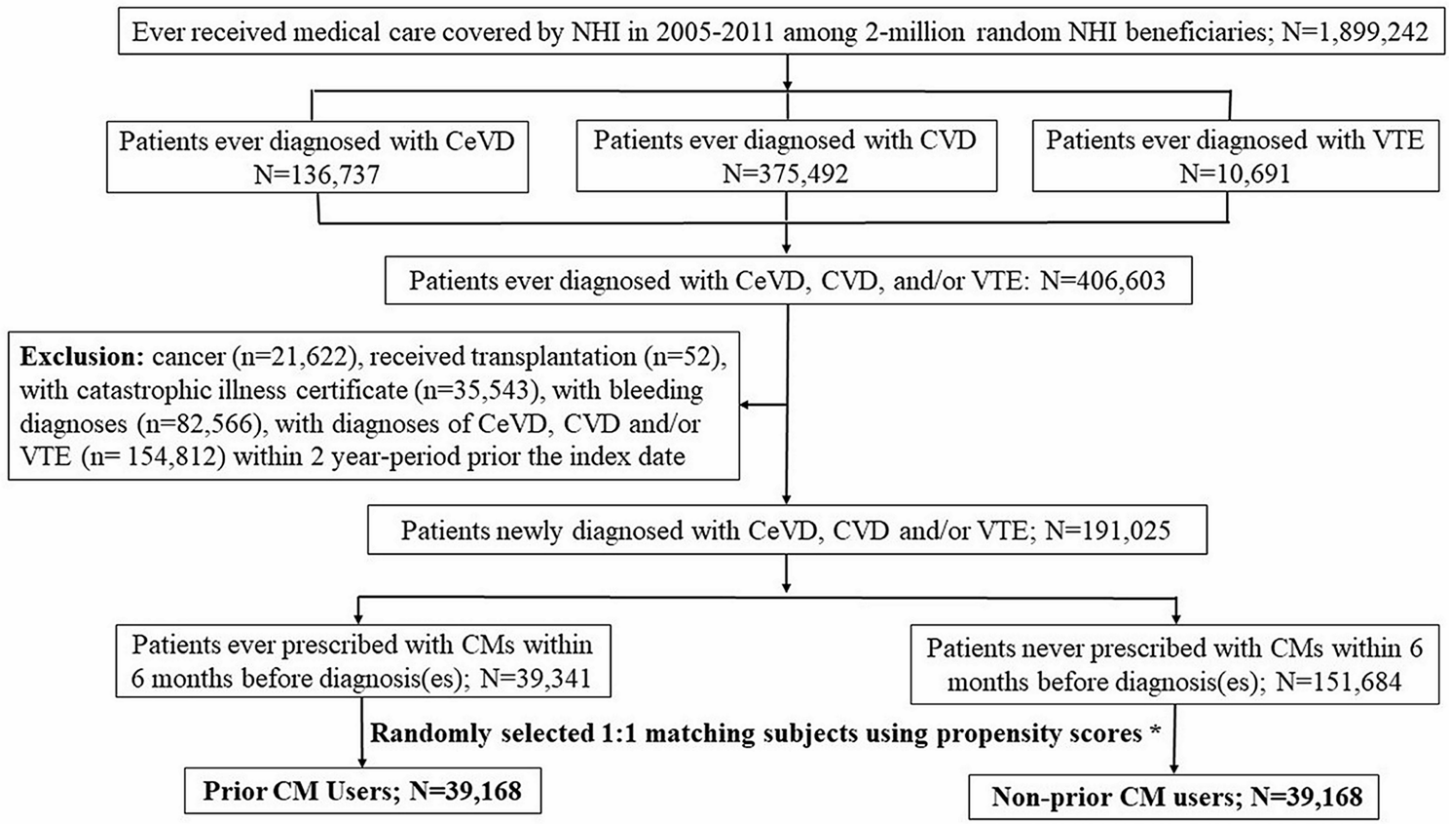
Flowchart of the study participant selection. CeVD = Cerebrovascular disease; CVD = Cardiovascular disease; VTE = Venous thromboembolism CMs = Chinese medications (extract granule of Chinese medications). *Using the following factors to perform propensity score to match: sex, age, group, national health insurance enrollment premium group and enrollment areas, urban level, prior related medications or comorbidities, which contributed the statistical differences between those prior exposure to CM or not

There were 191,025 patients with newly diagnosed CVD, CeVD, or VTE between 2005 and 2011. Of these patients, 39,351 (20.60%) were ≥ 65 years old and 110,221 (57.70%) were women. We found that 39,341 (20.59%) patients had received CMs and 151,684 patients (79.41%) had never received CMs within the 2-year period prior to the index date. After propensity score matching, we selected 39,168 patients in each group, namely prior and non-prior CM users, for further analysis. Figure 1 presents the flowchart of the study participant selection. The baseline characteristics, including residential area and disease and medication history, were all balanced between these two groups (all SMDs indicated a practically irrelevant difference; Table 1). The most common comorbidity was ulcer disease (~ 19%), followed by chronic obstructive pulmonary disease (~ 14%) and diabetes (~ 12%). The most commonly prescribed WMs were non-steroidal anti-inflammatory drugs (~ 63%), CVD drugs (e.g., anti-hypertensive medications [~ 36%]), and H2 blockers or proton pump inhibitors (~ 20%).

Of all patients first prescribed CMs after being newly diagnosed with CVD, CeVD, or VTE, the common indications included acute nasopharyngitis (common cold), cough, headache, lumbago (low back pain), and sleep disturbances (Table 2). The listed indications endorsed by TCM physicians were different between prior CM users (i.e., unspecified cardiac dysrhythmia, unspecified hypertensive heart and renal disease without mention of congestive heart failure or renal failure, and bronchitis) and non-prior CM users (sciatica, pain in a joint, multiple sites, and lumbar sprains and strains).

**Table 2:** The top 20 most frequent diagnoses for those patients first prescribed with Chinese medicinal formulas during 6-month periods after being diagnosed with new-onset cerebrovascular- or cardiovascular-related diseases

Ranking	All	Prior CM users	Non-prior CM users
1	Acute nasopharyngitis [common cold]	Acute nasopharyngitis [common cold]	Acute nasopharyngitis [common cold]
2	Cough	Cough	Cough
3	Headache	Headache	Headache
4	Lumbago (low back pain)	Lumbago (low back pain)	Other sleep disturbances
5	Other sleep disturbances	Other sleep disturbances	Lumbago (low back pain)
6	Sleep disturbances, unspecified	Sleep disturbances, unspecified	Sleep disturbances, unspecified
7	Palpitations	Palpitations	Myalgia and myositis, unspecified
8	Unspecified functional disorder of stomach	Unspecified functional disorder of stomach	Palpitations
9	Myalgia and myositis, unspecified	Constipation	Unspecified functional disorder of stomach
10	Constipation	Allergic rhinitis causes unspecified	Neuralgia, neuritis and radiculitis, unspecified
11	Allergic rhinitis cause unspecified	Myalgia and myositis, unspecified	Constipation
12	Dyspepsia and other specified disorders of function of stomach	Dyspepsia and other specified disorders of function of stomach	Allergic rhinitis cause unspecified
13	Neuralgia, neuritis and radiculitis, unspecified	Flatulence, eructation, and gas pain	Dyspepsia and other specified disorders of function of stomach
14	Flatulence, eructation, and gas pain	Neuralgia, neuritis and radiculitis, unspecified	Flatulence, eructation, and gas pain
15	Dizziness and giddiness	Dizziness and giddiness	Dizziness and giddiness
16	Cardiac dysrhythmia, unspecified	**Cardiac dysrhythmia**,** unspecified**	Essential hypertension, unspecified
17	Essential hypertension, unspecified	**Unspecified hypertensive heart and renal disease without mention of congestive heart failure or renal failure**	**Sciatica**
18	Bronchitis, not specified as acute or chronic	Essential hypertension, unspecified	**Pain in joint**,** multiple sites**
19	Unspecified hypertensive heart and renal disease without mention of congestive heart failure or renal failure	**Bronchitis**,** not specified as acute or chronic**	**Sprains and strains of lumbar**
20	Acute gastritis, without mention of hemorrhage	Acute gastritis, without mention of hemorrhage	Acute gastritis, without mention of hemorrhage

Ranking: 1 = most frequent diagnosis 20 = the 20th frequent diagnose; CM = Chinese medication (in terms of extract granules of Chinese medications); those indications being highlighted were different between Prior CM users and non-users

**Fig. 2:**
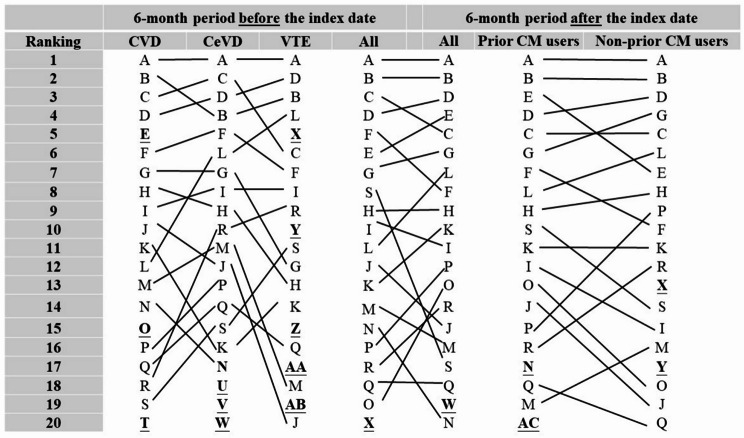
Ranking of the top 20 most frequently prescribed Chinese medicinal formulas during 6-month periods before or after being diagnosed with new-onset cerebrovascular- or cardiovascular-related diseases. Ranking where 1 = most frequently prescribed Chinese medicinal formulas and 20 = the 20th frequent Chinese medicinal formulas; CVD = Cardiovascular disease; CeVD = Cerebrovascular disease; VTE = Venous thromboembolism; CM = Chinese medication; A = Shu-Jing-Huo-Sie-Tang (SJHST); B = Jia-Wei-Siao-Yao-San (JWSYS); C = Ge-Gen-Tang (GGT); D = Shao-Yao-Gan-Cao-Tang (SYGCT); E = Jhih-Gan-Cao-Tang (JGCT); F = Chuan-Cyong-Cha-Tiao-San (CCCTS); G = Sie-Fu-Jhu-Yu-Tang (SFJYT); H = Ban-Sia-Sie-Sin-Tang (BSSST); I = Yin-Ciao-San (YCS); J = Ma-Sing-Gan-Shih-Tang (MSGST); K = Tian-Wang-Bu-Sin-Dan (TWBSD); L = Du-Huo-Ji-Sheng-Tang (DHJST); M = Siao-Chai-Hu-Tang (SCHT); N = Sin-Yi-Cing-Fei-Tang (SYCFT); O = Sheng-Mai-Yin (SMY); P = Suan-Zao-Ren-Tang (SZRT); Q = Gan-Lu-Yin (GLY); R = Liou-Wei-Di-Huang-Wan (LWDHW); S = Ping-Wei-San (PWS); T = Siao-Cing-Long-Tang (SCLT); U = Ling-Guei-Jhu-Gan-Tang (LGJGT); V = Zhi-Sou-San (ZSS); W = Long-Dan-Sie-Gan-Tang (LDSGT); X = Dang-Guei-Nian-Tong-Tang (DGNTT); Y = Shen-Tong-Zhu-Yu-Tang (STZYT); Z = Jhih-Bo-Di-Huang-Wan (JBDHW); AA = Mai-Men-Dong-Tang (MMDT); AB = Sang-Jyu-Yin (SJY); AC = Tian-Ma-Gou-Teng-Yin (TMGTY)

Among the entire cohort (*N* = 191,025), we observed a marked increase in CM use following the diagnosis of new-onset CeVD, CVD, or VTE, increasing from 20.60% before the diagnosis to 51.93% after the diagnosis (data not shown). Overall, there was a prominent shift in rankings of CM formulas and single CMs prior to and after a new diagnosis of CVD, CeVD, or VTE. The prior and non-prior CM users exhibited different prescription patterns in the 6 months preceding the index date (Fig. 2). Shu-Jing-How-Shiee-Tang (SJHST), coded as A, remained the most prescribed CM formula both before and after a new diagnosis of CVD, CeVD, or VTE. The prior prescription rate of CM formulas was 15.42% among all patients with newly diagnosed CVD, CeVD, or VTE (Table S2), and 13.80% and 12.77% for those who were diagnosed with CVD and CeVD, respectively (Table S4). Jia-Wei-Shiau-Yau San (JWSYS, coded as B) was also prescribed frequently for patients with newly diagnosed CVD, CeVD, or VTE. The prescription rate was 14.05% for those diagnosed with cardiac or vascular-related diseases (Table S2) and 10.42% for those diagnosed with CeVD (Table S4). Some CM formulas were prescribed specifically for patients with newly diagnosed CVD before and/or after the diagnosis (e.g., Jhih-Gan-Cao-Tang [JGCT], coded as E), Sheng-Mai- Yin [SMY], coded as O), Siao-Cing-Long-Tang [SCLT], coded as T) compared with patients with newly diagnosed CeVD and/or VTE.

Yan Hu Suo (YHS, coded as a) and Dan Shen (DS, coded as b) were the two most frequently prescribed single CMs, with a prescription rate of 14.40% and 14.30%, respectively, during the 6 months preceding the index date (Fig. S2). A few single CMs were unique for patients with newly diagnosed CVD, CeVD, or VTE, or for all patients before and/or after the diagnosis, compared with the other commonly used single CMs (e.g., Siang Fu [SF], coded as p; Hou Pu [HP], coded as n; Yuh Jin [YJ], coded as q; Yu Sing Cao [YSC], coded as r; Suan Zao Ren [SZR], coded as s; and Jhih Ke [JK], coded as t). These were prescribed more frequently for patients with CVD compared with patients diagnosed with CeVD and/or VTE.

Table 3 shows no differences in medication use between the prior and non-prior CM users in the 6 months following the index date, except for the exposure to CMs (73.51% vs. 30.34%, SMD 0.96; 51.90% among all eligible patients after propensity score matching). There was no significant difference between the two groups regarding all-cause mortality (2.67% vs. 3.84%, SMD − 0.07); all-cause severe CVD, CeVD, or VTE-related morbidity (33.81% vs. 31.97%, SMD 0.04); severe CVD or CeVD events (19.32% vs. 19.24%, SMD 0); revascularizations (2.34% vs. 2.62%, SMD − 0.02); and severe bleeding (18.32% vs. 16.57%, SMD 0.05) in the period of 6 months to 2 years after the index date.

**Table 3:** Comparisons of co-morbidities, medication use and relevant outcomes during 6-month or up to 2-year follow-up period for those patients who were prior CM users or not after being matched with propensity score among those patients being diagnosed with new-onset cerebrovascular- or cardiovascular-related diseases

Comparisons	After propensity score matching		
	Prior CM users (*n* = 39,168)	Non-prior CM users (*n* = 39,168)	SMD
**Co-morbidities (within 6-month follow-up period after the index date)**			
Acquired Immunodeficiency Syndrome	9 (0.02%)	10 (0.03%)	-0.01
Chronic obstructive pulmonary disease	5134 (13.11%)	4663 (11.91%)	0.04
Connective tissue disease	1154 (2.95%)	806 (2.06%)	0.06
Dementia	510 (1.30%)	603 (1.54%)	-0.02
Diabetes	4750 (12.13%)	4761 (12.16%)	0
Diabetes with end organ damage	2171 (5.54%)	1960 (5.00%)	0.02
Hemiplegia	599 (1.53%)	350 (0.89%)	0.06
Mild liver disease	740 (1.89%)	665 (1.70%)	0.01
Moderate/severe liver disease	62 (0.16%)	41 (0.10%)	0.02
Moderate/severe renal disease	635 (1.62%)	608 (1.55%)	0.01
Peripheral vascular disease	585 (1.49%)	549 (1.40%)	0.01
Ulcer disease	7224 (18.44%)	6237 (15.92%)	0.07
**Medication use (within 6-month follow-up period after the index date)**			
CMs	28,793 (73.51%)	11,884 (30.34%)	0.96
CVD/CeVD drugs^a^	25,735 (65.70%)	26,365 (67.31%)	-0.03
DM drugs	4664 (11.91%)	4836 (12.35%)	-0.01
GI protectors	15,316 (39.10%)	14,180 (36.20%)	0.06
Non-steroidal anti-inflammatory drugs	33,431 (85.35%)	32,942 (84.10%)	0.03
SSRIs/SNRIs	2331 (5.95%)	1831 (4.67%)	0.06
Steroids	13,881 (35.44%)	12,929 (33.01%)	0.05
VTE drugs	475 (1.21%)	519 (1.33%)	-0.01
**Secondary cardiac or vascular-related events (up to 2-year follow-up period after the index date)**			
All-cause mortality	1045 (2.67%)	1505 (3.84%)	-0.07
All-cause severe CVD, CeVD, or VTE related morbidities	13,243 (33.81%)	12,523 (31.97%)	0.04
Severe CeVD events	7569 (19.32%)	7534 (19.24%)	0
Revascularizations	917 (2.34%)	1025 (2.62%)	-0.02
Severe bleeding	7177 (18.32%)	6492 (16.57%)	0.05
CVD in OPD	20,092 (51.30%)	19,111 (48.79%)	0.05
CeVD in OPD	4301 (10.98%)	4768 (12.17%)	-0.04
VTE in OPD	309 (0.79%)	266 (0.68%)	0.01

^a^ abciximab, acebutolol, acetylsalicylic acid, adenosine, amiloride, amiodarone, amlodipine, ascorbic acid, aspirin, atenolol, atorvastatin, betaxolol, bezafibrate, bisoprolol, bumetanide, candesartan, captopril, carvedilol, cholestyramine, cholestyramine, cilazapril, cilostazol, clofibrate, clonidine, clopamide, clopidogrel, colestipol, diazoxide, digoxin, diltiazem, disopyramide, doxazosin, enalapril, enoxaparin, eptifibatide, esmolol, ezetimibe, felodipine, fenofibrate, flecainide acetate, fluvastatin, fosinopril, furosemide, gemfibrozil, hydralazine, hydrochlorothiazide, imidapril, irbesartan, isosorbide, labetalol, lacidipine, lercanidipine, lidocaine, lisinopril, losartan potassium, lovastatin, methyldopa, metoprolol, mexiletine, minoxidil, nadolol, niacin, nicardipine, nicorandil, nifedipine, nimodipine, nitroglycerin, nitroglycerin, nitroprusside, olmesartan, pentoxifylline, perindopril, phentolamine, pindolol, pitavastatin, plasminogen activator recombinant human tissue-typ, pravastatin, prazosin, probucol, procainamide, propafenone, propranolol, urokinase, quinapril, quinidine, ramipril, rosuvastatin, simvastatin, sotalol, spironolactone, streptokinase, telmisartan, ticlopidine, tirofiban, tocopherol, triamterene, trichlormethiazide, valsartan, verapamil

CMs = Chinese medications (in terms of extract granules of Chinese medications); DM = Diabetes mellitus; CVD = Cardiovascular disease; CeVD = Cerebrovascular disease; VTE = Venous thromboembolism; GI = Gastrointestinal; SSRI = Selective serotonin receptor inhibitors, SNRI = Serotonin–norepinephrine reuptake inhibitors; OPD = Outpatient department

## Discussion

To the best of our knowledge, this is the first comprehensive study to examine patterns of TCM use—specifically prepared as extract granules (in terms of CMs)—including use prior to diagnosis, concurrent WM use, and outcome assessments among patients with newly diagnosed cardiac or vascular-related diseases. More than 30% of patients with newly diagnosed cardiac or vascular-related diseases began using CMs, while 73.51% of previous users maintained their usage. SJHST and JWSYS were the two most frequently prescribed CM formulas, regardless of whether they were prescribed prior to or after the diagnosis of new-onset cardiac or vascular-related diseases. Acute nasopharyngitis, cough, headache, low back pain, and sleep disturbances were the top 5 diagnoses for which TCM physicians prescribed these CMs. There were no significant differences between prior and non-prior CM users regarding comorbidities, the medication patterns, and the secondary cardiac or vascular-related events after being diagnosed with new-onset cardiac or vascular-related diseases. However, the notable incidence of secondary events and major bleeding underscore the importance of monitoring the disease progression and bleeding risk in both groups.

In TCM, syndrome differentiation (Zheng) involves a holistic assessment of a patient’s condition through the integration of various diagnostic elements (e.g., signs, symptoms, pulse characteristics, tongue appearance, and other clinical indicators) [5]. Rather than target a specific disease or symptom—as is common in Western medicine—TCM practitioners evaluate patterns of internal imbalance within the body’s functional systems. Based on this assessment, they determine a personalized treatment plan that may include CM, acupuncture, and/or other modalities, aiming to restore systemic harmony and address the underlying cause of the condition [22]. Although the NHIRD used in our study does not include specific Zheng classifications, it does provide disease diagnoses based on the International Classification of Diseases, Ninth Revision (ICD-9) or ICD-10 codes. We acknowledge that syndrome differentiation is an intrinsic aspect of TCM, which may influence treatment strategies even for patients who share the same Western diagnosis [22]. In our dataset, the most commonly associated diagnoses among prior CM users included acute nasopharyngitis, cough, headache, low back pain, and sleep disturbances. These accompanying conditions may also contribute to syndrome differentiation and, consequently, variations in prescriptions of CMs (either extract granules, pills, or decoction formats). For example, two patients diagnosed with ischemic stroke might receive different CMs depending on their individualized Zheng patterns (e.g., one presenting with “wind-phlegm obstructing the channels” versus another with “yin deficiency and internal wind”), particularly if they also present with one or more of the aforementioned five common conditions during their visits to TCM physicians.

The finding that SJHST was the most commonly prescribed CM formula across different diseases and exposure periods or timings among patients with new-onset cardiac or vascular-related conditions differs from previous studies that have focused on stroke [12], atrial fibrillation [23], and ischemic heart disease [24]. Importantly, the approach we used to estimate the use of CMs is different from these previous studies (e.g., the percentage of all patients with cardiac or vascular-related diseases or the number of person-days) (Table S4). In prior studies that also utilized the NHIRD (Table S4), SJHST was the fifth most prescribed CM formula for stroke patients [12], the seventh most prescribed CM formula for patients with atrial fibrillation [23], and was not in the top 10 for patients with ischemic heart disease [24]. Interestingly, SJHST was the most prescribed CM formula for patients receiving anticoagulant drugs during the period of 2000–2013 and was associated with a lower risk of major bleeding events [7]. Based on TCM theory, SJHST, which comprises 17 single CMs (including *Paeoniae Radix* [*Paeonia lactiflora*] and *Angelica sinensis Radix* [*Danggui*, *A. sinensis*]) is a blood-rectifying formula that soothes menstruation, activates blood, and dispels wind [18]. It is frequently used for patients “with thrombosis-associated pain and osteoarthritis.” The concurrent use of SJHST and warfarin prolongs bleeding times and anticoagulant effects in animal studies [25, 26], but SJHST seemed likely to reduce the bleeding tendency among patients [7]. However, treating patients with cardiac or vascular-related diseases using antithrombotic agents (e.g., anticoagulants and antiplatelet agents) poses profound challenges in balancing the risks of re-thromboembolism and bleeding. These risks may be further complicated by the concurrent use of various combinations of TCM [27, 28]. Therefore, healthcare providers—including physicians and pharmacists—should collaborate closely to carefully evaluate the risk-benefit profiles of using Chinese medicine alongside WMs in this patient population.

JWSYS was the second most commonly prescribed CM formula among all eligible patients in the present study. This finding is similar to another study based on 2004 data from the NHIRD [27]. JWSYS contains Dang Gui, which might potentially increase the anticoagulant activity of aspirin/clopidogrel, but there was no significant risk of bleeding in an animal study [28]. Further, both Yan Hu Suo and Dan Shen were listed as the first or second most prescribed single CMs in a recent study using the NHIRD [7]. The combination of Dan Shen and clopidogrel may have synergic effects of bleeding [29]. Clinical practitioners should be aware of these interactions between WMs and CMs to balance the benefits and risks of using combination therapies for patients with cardiac or vascular-related diseases.

We found that prior CM use relative to a diagnosis of new-onset CVD, CeVD, or VTE may not significantly impact follow-up medication use, co-morbidities, and recurrent cardiac or vascular-related events, including mortality and severe bleeding. Other studies have reported different results when focusing on patients with newly diagnosed stroke [12], patients with atrial fibrillation and using warfarin [23], and patients with prevalent ischemic heart disease [24]. Those authors claimed that the use of CMs leads to positive effects. The results should be interpreted with caution because the authors of those studies used various approaches to define the exposure to CMs (including different types of CMs, timing, the corresponding diagnoses, and/or estimations of exposure), and they did not provide information related to the concurrent use of WMs. Future studies investigating the effectiveness of different types of CMs (combinations of different single CMs [Dan fang], combinations of CM formulas with various dosages [Fu fang], various regimens of CM formulas with fixed or individualized dosages in line with classical or well-known TCM textbooks [Fang ji], and the classical formula combined with a few single CMs [Chia chien fang] based on the TCM theories) for patients with cardiac or vascular-related diseases may be needed [24].

There was an increasing tendency to use CMs before or after a new diagnosis of cardiac or vascular-related diseases (~ 20% among all eligible patients to over 50% among all matched patients, or 73.51% vs. 30.34% among those who were prior and non-prior CM users). Such a trend is consistent with the increased use of CMs in previous studies [27, 30, 31]. Although there are some concerns regarding drug interactions between CMs and WMs in the context of vascular-related diseases [25], the benefits (e.g., reduced mortality and/or improved quality of life) of using CMs with or without WMs appear to outweigh the risks and likely to contribute the increased use of TCM [7–9, 32]. Additional explorations with a more rigorous causal inference framework are needed to determine whether CMs provide additional therapeutic value for patients at high tendency of recurrent of cardiac or vascular-related diseases while balancing the concerns of potential adverse effects.Although we did not find significant differences in key outcomes—such as secondary events and mortality—between the two groups, the clinical implications, including higher bleeding rates among CM users with or without WMs, may warrant careful clinical consideration in clinical practice.

Our study has six main limitations. First, there are no specific diagnostic codes whenever TCM physicians prescribe CMs covered by the NHI. TCM treatments are usually decided based on the TCM theories, for examples differentiation of various Zheng (in terms of syndromes) for patients but not for the symptoms of the diseases [5] and characteristics of CMs [6]. The corresponding top 20 diagnoses and prescribed CM formulas and single CMs were different from the personalized approaches of CM remedies (or regimens) based on the specific Zheng of TCM theories for individual patients. Of note, the new ICD-11 has been in effect since 2022 and includes codes related to disorders and patterns in TCM terminology [33] in Taiwan. Second, we only focused on CMs covered by the NHI during 2003–2013. Further, we did not consider decoction of crude CM materials, over-the-counter with CMs, or dietary supplement products with CMs in this study. We assumed that the percentages of patients using decoctions of crude CM materials may be equally balanced between the prior and non-prior CM users. Third, we did not consider residual confounding that might be associated with the use of CM prior to or after a diagnosis of new-onset cardiac or vascular-related diseases due to the limitations of the NHIRD. Nevertheless, we selected 25 covariates and used propensity score matching to adjust for baseline disease severity, and we believe that such effect was comparable. Future randomized controlled trials should consider incorporating detailed measures of CM doses or disease severity to address this. Moreover, we conducted a power analysis to evaluate the ability to detect the specified alternative hypothesis for matched dichotomous outcomes. With a sample size of 39,168 matched pairs (1:1), assuming an exposure probability of 0.2 among controls and a hypothetical odds ratio of 0.8, the analysis achieved over 99% statistical power at a significance level (α) of 0.05. This supports the robustness and validity of our findings. Fourth, our study was cross-sectional rather than longitudinal. We assumed that secondary cardiac or vascular-related events or outcomes after new-onset CVD, CeVD, or VTE are associated with prior use of CMs with and without relevant WMs. Finally, our analysis may have underestimated or overestimated the secondary cardiac or vascular-related events due to the 2-year follow-up period being adopted. However, we compared and contrasted the patterns of CM formulas, single CMs, and the diagnoses corresponding to CM prescriptions after performing propensity score matching for the baseline characteristics and prior disease and medication use history. There were no differences between the prior and non-prior CM users regarding the co-morbidities and WM patterns, as well as the secondary cardiac or vascular-related events, after being diagnosed with new-onset cardiac or vascular-related diseases. Nevertheless, additional in-depth explorations to assess the impact of concurrent use of CMs with WMs on the follow-up outcomes and further assessment of the potential risk-benefit ratios of CM use are needed.

## Conclusions

This population-based cohort study establishes a foundation for assessing prescription patterns of CMs among patients following a new diagnosis of cardiac or vascular-related diseases. Over 30% of patients with newly diagnosed cardiac or vascular-related diseases initiated the use of CMs, whereas 73.51% of individuals with prior exposure continued their use during the follow-up period. SJHST and JWSYS were the two most frequently prescribed CM formulas, irrespective of usages before the index date. The significant incidence of secondary cardiac or vascular-related events and major bleeding among patients with new-onset cardiac or vascular-related diseases, whether or not they had used CMs prior to the diagnosis, underscores the importance of careful monitoring by healthcare professionals. Evaluating the risk–benefit balance for patients using CMs alongside prescribed WMs remains crucial. Further research is needed to assess the potential advantages and risks of combining CMs with WMs in patients at high risk of cardiac or vascular-related conditions.

## Electronic supplementary material

Below is the link to the electronic supplementary material.

Supplementary Material 1

## Data Availability

The deidentified retrospective datasets used and/or analyzed during the current study are available from the first author (H.-W. Lin) under the reasonable request.
