# Supplementary material for: Prescription patterns of traditional Chinese medications and potential consequences in patients with new-onset cardiac or vascular-related diseases: a nationwide cohort study
Source: BMC Complement Med Ther. 2025 Jul 2;25:216. doi: 10.1186/s12906-025-04945-4 (PMC12217369; doi:10.1186/s12906-025-04945-4)
Supplement: Supplementary file 1 — Supplementary Material 1 [file 12906_2025_4945_MOESM1_ESM.docx]

Supplements


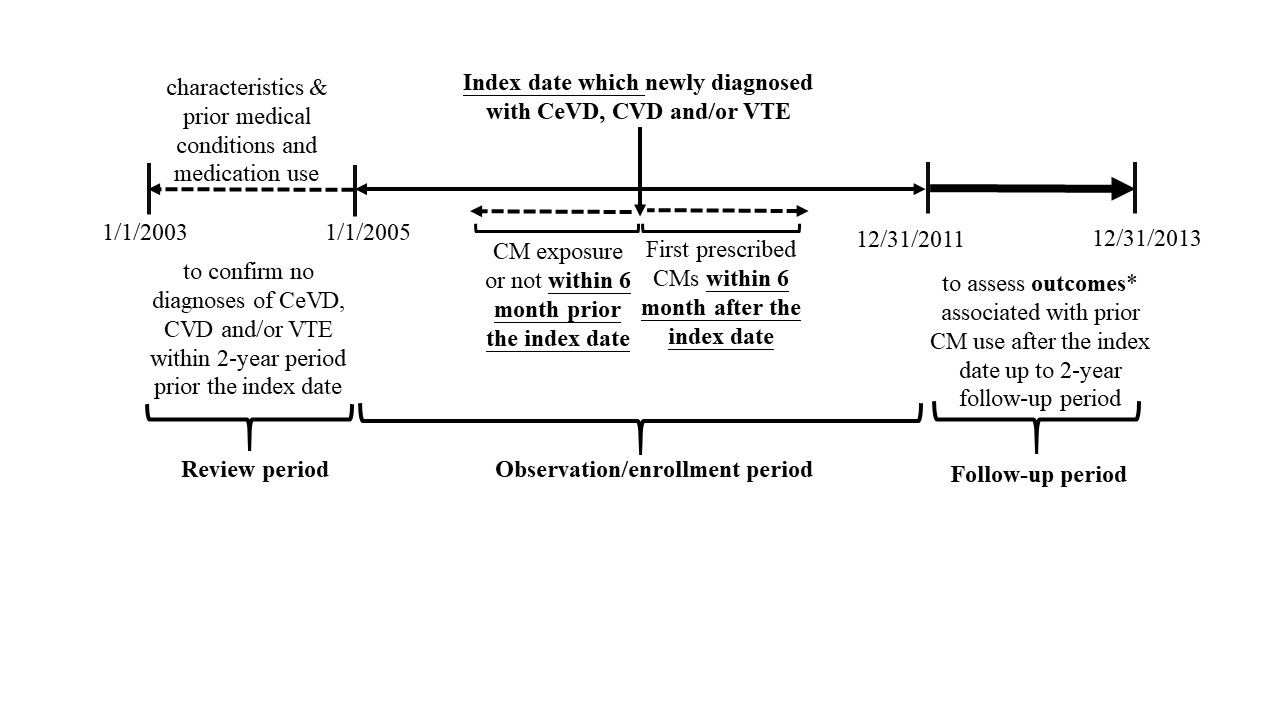


**Supplement Figure 1: Study design and periods to collect relevant data**

CeVD= Cerebrovascular disease; CVD= Cardiovascular disease; VTE= Venous thromboembolism CMs=Chinese medications (in terms of extract granules of Chinese medications)

**Supplement Table 1 List of diseases and/or diagnoses of interest**

| Disease | The International Classification of Diseases, 9th Revision, Clinical Modification |
| --- | --- |
| Cancer | 140,141,142,143,144,145,146,147,148,149,150,151,152,153,154,155,156,157,158,159,160, 161,162,163,164,165,166,167,168,169,170,171,172,173,174,175,176,177,178,179,180,181, 182,183,184,185,186,187,188,189,190,191,192,193,194,195,196,197,198,199,200,201,202, 203,204,205,206,207,208 |
| Cardiovascular disease | 393,394,395,396,397,398,402,403,404,410,411,412,413,414,425,427,428,429,440 |
| Cerebrovascular disease | 430,431,432,433,434,435,436,437,438,7814,7843,9970,381,384 |
| Major bleeding | 430,431,4590,4320,4321,4329,4560,5307,5693,5780,5781,5789,5967,5997,6265,6266,6268, 6269,6270,6271,7847,7848,7863,36043,36361,36362,37272,37923,45620,53082,53100, 53101,53120,53121,53140,53141,53160,53161,53200,53201,53220,53221,53240,53241, 53260,53261,53300,53301,53320,53321,53340,53341,53360,53361,53400,53401,53420, 53421,53440,53441,53460,53461,53501,53511,53521,53531,53541,53551,53561,53783, 56202,56203,56212,56213,56881,56985,71910,71911,71912,71913,71914,71915,71916, 71917, 71918,71919,99811, 99812 |
| Peripheral vascular diseases | 441,442,4431-4439,4471,7854,3813,3814,3816,3818,3833,3834,3836,3838,3843,3844,3846, 3848,3922–3926,3929 |
| Transplantation | v420,v427,v421,v426,5569,5051,5052,5053,5054,5055,5056,5057,5058,5059,3350,3351, 3352,375 |
| Ulcer disease | 531,532,533,534 |
| Venous thromboembolism | 4511,4512,4531,4532,4534,4538,4539,415,45183 |

**Supplement Table 2: List of most frequently prescribed Chinese medication formulas during 6-month periods before being diagnosed with new-onset cerebrovascular- or cardiovascular-related diseases**

| **Code** | **English name** | **Abbreviation** | **Classification of formula** | **Efficacy upon Chinese medicine theory** | **% of 35,162 patients with newly diagnosed vascular-related diseases during 6-month period before the index date** |
| --- | --- | --- | --- | --- | --- |
| A | Shu-Jing-Huo-Sie-Tang | SJHST | Blood-rectifying | Soothe menstruation, activate blood, and dispel wind. | 15.42 |
| B | Jia-Wei-Siao-Yao-San | JWSYS | Harmonizing | Soothe the liver, release depression, clear heat to cool the blood. | 14.05 |
| C | Ge-Gen-Tang | GGT | Exterior-Resolving | Promote sweating to release the flesh. | 12.70 |
| D | Shao-Yao-Gan-Cao-Tang | SYGCT | Harmonizing | Relax tension to relieve pain. | 12.21 |
| E | Jhih-Gan-Cao-Tang | JGCT | Dryness-Moistening | Tonify qi and blood, enrich yin, and rehabilitate vessel. | 10.09 |
| F | Chuan-Cyong-Cha-Tiao-San | CCCTS | Exterior-Resolving | Dispel wind and relieve pain. | 10.97 |
| G | Sie-Fu-Jhu-Yu-Tang | SFJYT | Blood-Rectifying | Activate blood and resolve stasis, and move qi to relieve pain | 9.59 |
| H | Ban-Sia-Sie-Sin-Tang | BSSST | Harmonizing | Harmonize the stomach to descend counterflow. | 9.09 |
| I | Yin-Ciao-San | YCS | Exterior-resolving | Outthrust through the exterior with pungent-cool, and clear heat and detoxicate. | 9.08 |
| J | Ma-Sing-Gan-Shih-Tang | MSGST | Exterior-Resolving | Diffuse with pungent-cool, and clear the lung to calm panting. | 8.54 |
| K | Tian-Wang-Bu-Sin-Dan | TWBSD | Spirit-Quieting | Calm the spirit, transform phlegm and drool, dispel vexing heat, as well as enriching yin and nourishing the blood. | 8.23 |
| L | Du-Huo-Ji-Sheng-Tang | DHJST | Dampness-Dispelling | Dispel wind-dampness, resolve painful impediment, tonify qi and blood. | 8.60 |
| M | Siao-Chai-Hu-Tang | SCHT | Harmonizing | Harmonize and release the lesser yang. | 8.04 |
| N | Sin-Yi-Cing-Fei-Tang | SYCFT | Heat-Clearing | Clear lung heat. | 7.92 |
| O | Sheng-Mai- Yin | SMY | Supplementing | Replenish original qi damaged by heat with shortness of breath, fatigue, thirst, and profuse sweating, as well as lung vacuity with cough. | 7.17 |
| P | Suan-Zao-Ren-Tang | SZRT | Spirit-Quieting | Nourish the blood and quiet the spirit; clear heat and eliminate vexation. | 7.63 |
| Q | Gan-Lu-Yin | GLY | Damp-heat‒Clearing | Nourish yin and clear dampness-heat | 7.31 |
| R | Liou-Wei-Di-Huang-Wan | LWDHW | Supplementing | Nourish yin to tonify the kidney. | 7.42 |
| S | Ping-Wei-San | PWS | Dampness-resolving | Dry dampness to fortify the spleen, regulate qi and harmonize the middle. | 9.14 |
| T | Siao-Cing-Long-Tang | SCLT | Exterior-Resolving | Release the exterior to dissipate cold, warm the lung, and resolve fluid retention. | 6.03 |
| U | Ling-Guei-Jhu-Gan-Tang | LGJGT | Dampness-Disinhibiting | Fortify the spleen, drain dampness, and resolve phlegm. | 5.33 |
| V | Zhi-Sou-San | ZSS | Exterior-Resolving | Relieve coughs, resolve phlegm, soothe the lungs, and ease symptoms | 5.61 |
| W | Long-Dan-Sie-Gan-Tang | LDSGT | Heat-clearing fire-draining | Heat-clearing fire-draining formula | 6.91 |
| X | Dang-Guei-Nian-Tong-Tang | DGNTT | Dampness-Resolving | Clear heat and dry dampness, activate blood to relieve pain. | 4.50 |
| Y | Shen-Tong-Zhu-Yu-Tang | STZYT | Blood-Rectifying | Promote blood circulation and qi, remove blood stasis, unblock collaterals, relieve numbness, and relieve pain. | 4.24 |
| Z | Jhih-Bo-Di-Huang-Wan | JBDHW | Supplementing | Nourish yin to suppress fire. | 5.27 |
| AA | Mai-Men-Dong-Tang | MMDT | Dryness-Moistening | Clear and nourish the lungs, stomach and lower qi. | 5.09 |
| AB | Sang-Jyu-Yin | SJY | Exterior-Resolving | Disperse wind to clear heat, diffuse the lung to suppress cough. | 5.43 |
| AC | Tian-Ma-Gou-Teng-Yin | TMGTY | Wind-Controlling | Alleviate headache, dizziness, tinnitus, and flowery vision (dizzy vision) due to liver wind stirring internally. | 5.24 |

**Supplement Table 3: List of most frequently prescribed single Chinese medication during 6-month periods before being diagnosed with new-onset cerebrovascular- or cardiovascular-related diseases**

| Code | English name | Abbreviation | Official name (Latin) | Classification of material | Efficacy upon Chinese medicine theory | % of 35,162 patients newly diagnosed with CVD/CeVD/VTE during 6-month period before the index date |
| --- | --- | --- | --- | --- | --- | --- |
| a | Yan Hu Suo | YHS | Corydalis Rhizoma | Blood-regulating medicinal (Blood-activating and stasis-dispelling medicinal) | Activate blood, move qi, relieve pain, regulate menstruation. | 14.40 |
| b | Dan Shen | DS | Salviae Miltiorrhizae Radix et Rhizoma | Blood-regulating medicinal (Blood-activating and stasis-dispelling medicinal) | Activate blood and eliminate stasis, regulate menstruation to relieve pain, cool the blood to disperse abscesses, eliminate vexation, and calm mental state. | 14.30 |
| c | Jie Geng | JG | Platycodonis Radix | Phlegm-dispelling medicinal (Heat-phlegm clearing and resolving medicinal) | Diffuse the lung, dispel phlegm, soothe throat, expel pus. | 11.40 |
| d | Ge Gen | GG | Puerariae Radix | Exterior-releasing medicinal (Pungent-cold exterior-releasing medicinal) | Promote sweating to release the exterior, engender fluid, outthrust rashes, act as antidiarrheal. | 11.20 |
| e | Huang Cin | HC | Scutellariae Radix | Heat-clearing medicinal (Heat-clearing and dampness-drying medicinal) | Clear heat, dry dampness, purge fire and detoxicate, cool the blood and stop bleeding, eliminate heat, and prevent miscarriage. | 10.20 |
| f | Chuan Bei Mu | CBM | Fritillariae Cirrhosze Bulbus | Phlegm-dispelling medicinal (Heat-phlegm clearing and resolving medicinal) | Clear heat to transform phlegm, moisten lungs to suppress cough, dissipate binds to alleviate edema. | 10.10 |
| g | Bai Jhih | BJ | Angelicae Dahuricae Radix | Exterior-releasing medicinal (Pungent-warm exterior-releasing medicinal) | Release the exterior to dissipate cold, dispel wind and eliminate dampness, disperse swelling and expel pus, open orifices, and relieve pain. | 8.90 |
| h | Gan Cao | GC | Glycyrrhizae Radix et Rhizoma | Tonifying and replenishing medicinal (Qi tonifying medicinal) | Supplement spleen and stomach to tonify qi, moisten the lung to suppress cough and dispel phlegm, relax tension to relieve pain, mitigate the sharp actions of other medicines, harmonize other medicines. | 7.50 |
| i | Du Jhong | DJ | Eucommiae Cortex | Tonifying and replenishing medicinal (Yang tonifying medicinal) | Tonify liver and kidney, strengthen sinew and bone, prevent abortion. | 7.10 |
| j | Ku Sing Ren | KSR | Armeniacae Semen Amarum\ | Phlegm-dispelling medicinal (Cough-suppressing and panting-calming medicinal) | Suppress cough and calm panting, moisten the intestine, and relax the bowel. | 7.10 |
| k | Mai Men Dong | MMD | Ophiopogonis Radix | Tonifying and replenishing medicinal (Yin tonifying medicinal) | Nourish yin and moisten lungs, calm cough, eliminate phlegm, supplement stomach and engender fluid, clear heart to eliminate vexation. | 7.10 |
| l | Huang Ci | HC | Astragali Radix | Tonifying and replenishing medicinal (Qi tonifying medicinal) | Tonify qi and upraise yang, defend qi to secure the exterior, expel toxin, and promote tissue regeneration, induce diuresis to alleviate edema. | 6.80 |
| m | Chuan Cyong | CC | Chuanxiong Rhizoma | Blood-regulating medicinal (Blood-activating and stasis-dispelling medicinal) | Activate blood and move qi, dispel wind to relieve pain. | 6.40 |
| n | Hou Pu | HP | Magnoliae Cortex | Dampness-Disinhibiting medicinal | Promote qi, remove dampness, eliminate accumulation, and relieve asthma. | 6.40 |
| o | Syuan Shen | SS | Scrophulariae Radix | Heat-clearing medicinal (Heat-clearing and blood-cooling medicinal) | Clear heat to cool the blood, nourish yin and detoxicate. | 6.20 |
| p | Siang Fu | SF | Cyperi Rhizoma | Qi-regulating medicinal | Regulate qi and release depression. | 5.80 |
| q | Yuh Jin | YJ | Curcumae Radix | Blood-regulating medicinal (Blood-activating and stasis-dispelling medicinal) | Activate blood circulation, promote qi, relieve pain and stagnation, clear the heart, promote yin, reduce jaundice, and cool blood. | 5.40 |
| r | Yu Sing Cao | YSC | Houttuyniae Herba | Heat-clearing medicinal | Clear heat and detoxicate, induce diuresis and relieve strangury, disperse abscesses, and expel pus. | 5.20 |
| s | Suan Zao Ren | SZR | ZIZIPHI SPINOSAE SEMEN | Tranquillizing medicinal (Heart-nourishing  tranquillizing medicinal) | Nourish the heart to tranquilize, relieve sweating  and generate fluid. | 5.10 |
| t | Jhih Ke | JK | CITRI FRUCTUS IMMATURUS | Qi-regulating medicinal | Move qi and harmonize the middle burner, resolve phlegm and  food. | 5.10 |
| u | Syu Duan | SD | Dipsaci Radix | Tonifying and replenishing medicinal (Yang- tonifying medicinal) | Tonify liver and kidney, regulate blood vein, strengthen sinews and bones | 1.20 |
| v | Chuan Niou Si | CNS | Cyathulae Radix | Blood-regulating medicinal (Blood-activating and stasis-dispelling medicinal) | Activate blood and eliminate stasis, promote menstruation, relieve pain. | 1.10 |
| w | Ji Sie Teng | JST | Spatholobi Caulis | Blood-regulating medicinal (Blood-activating and stasis-dispelling medicinal) | Move blood and tonify blood, relax sinews and activate collateral. | 1.10 |
| x | Ye Jiao Teng | YJT | Caulis Polygoni Multiflori | Spirit-Quieting medicinal | Nourish and calm the mind, unblock collaterals, and dispel wind. | 1.00 |
| y | Da Huang | DH | Rhei Radix ex Rhizoma | Purgative medicinal (Offensive purgative medicinal) | Remove accumulation with purgation, purge fire, clear heat and detoxicate, activate blood and eliminate stasis, clear heat, and drain dampness. | 1.00 |
| z | Mu Gua | MG | Chaenomelis Fructus | Dampness-dispelling medicinal (Wind-dampness dispelling medicinal) | Relax sinews and activate collateral, transform dampness to open stomach. | 0.20 |
| aa | Hwang Bor | HB | Phellodendri Cortex | Heat-clearing medicinal | Clear away heat and dampness, purge fire and detoxify, reduce fever, and remove steam. | 0.10 |
| ab | Yi Yi Ren | YYR | Coicis Semen | Dampness-dispelling medicinal (Dampness draining diuretic medicinal) | Fortify spleen and drain dampness, clear heat to expel pus, eliminate impediment and act as an antidiarrheal. | 0.10 |
| ac | Che Cian Zih | CCZ | Plantaginis Semen | Dampness-dispelling medicinal (Dampness draining diuretic medicinal) | Induce diuresis, relieve strangury, dispel wind and act as an antidiarrheal, clear liver to improve vision, clear lungs, and resolve phlegm. | 0.10 |
| ad | Bai Jhu | BJ | Atractylodis Macrocephalae Rhizoma | Tonifying and replenishing medicinal (Qi tonifying medicinal) | Tonify qi, fortify the spleen, dry dampness to induce diuresis, relieve sweating, and prevent abortion. | 0.10 |
| ae | Fu Ling | FJ | PORIA | Dampness-dispelling medicinal (Dampness draining diuretic medicinal) | Induce diuresis to drain dampness, fortify the  spleen and stomach calm the mind. | 0.10 |
| af | San Ci | SC | Notoginseng Radix et Rhizoma | Blood-regulating medicinal) | Dispel stasis and stop bleeding; activate blood to relieve pain. | <0.10 |
| ag | Hai Piao Siao | HPS | Sepiae Endoconcha | Astringent medicinal | Astringent, hemostatic, secure essence and stanch vaginal discharge, inhibit acidity to relieve pain, astringe moisture and promote wound healing. | <0.10 |
| ah | Mei Yao | MY | Myrrha | Blood-regulating medicinal (Blood-activating and stasis-dispelling medicinal) | Activate blood to relieve pain, disperse swelling and promote tissue regeneration. | <0.10 |
| aj | Ru Siang | RS | Olibanum | Blood-regulating medicinal (Blood-activating and stasis-dispelling medicinal) | Activate blood, move qi, relieve pain, disperse swelling and promote tissue regeneration. | <0.10 |

**Supplement Table 4: The top 10 most commonly prescribed Chinese medication formula for all eligible patients or those who were newly diagnosed with cardiovascular disease (CVD) or Cerebrovascular Disease (CeVD) in current study and stroke patients across different studies**

|  | **6-month prior to the newly diagnosis(es)** | | | |  | **First TCM service with CM after newly diagnosed with… (Chang, Lee et al. 2016)** | |  | **Randomly sampled cohort who visited TCM clinics and diagnosed with (n=4317) (Wang, Yen et al. 2022)** | |  | **Commonly used CHM products among CHMs users who newly diagnosed subjects with AF ≥ 20 years of age treated with warfarin between 1998 and 2007 (n=2670) (Zheng, Livneh et al. 2020)** | |
| --- | --- | --- | --- | --- | --- | --- | --- | --- | --- | --- | --- | --- | --- |
| **Ranking** | **CVD only** | **% of all CVD pts** | **CeVD only** | **% of all CeVD pts** |  | **Stoke** | **Number of person-days** |  | **IHD** | **% of CM prescription** |  | **AF** | **Frequency** |
| 1 | Shu-Jing-Huo-Sie-Tang | 13.80% | Shu-Jing-Huo-Sie-Tang | 12.77% |  | Bu-yang-huan-wu-tang | 59,034 |  | Zhi-Gan-Cao-Tang | 24.70% |  | Tao Hong SiWu Tang  (THSWT) | 7,877 |
| 2 | Jia-Wei-Siao-Yao-San | 13.30% | Ge-Gen-Tang | 10.42% |  | Xue-fu-Shu-yu-tang | 13,816 |  | Xue-Fu-Zhu-Yu-Tang | 21.30% |  | Xue Fu Zhu Yu Tang (XFZYT | 6,190 |
| 3 | Ge-Gen-Tang | 11.60% | Shao-Yao-Gan-Cao-Tang | 9.99% |  | Ma-zi-ren-wan | 13,450 |  | Sheng-Mai-Yin | 17.25% |  | Fu Yuan Huo Xue Tang  (FYHXT) | 6,140 |
| 4 | Shao-Yao-Gan-Cao-Tang | 11.00% | Jia-Wei-Siao-Yao-San | 9.44% |  | Tian-ma-gou-teng-yin | 12,365 |  | Gua-Lou-Xie-Bai-Ban-  Xia-Tang (Gualou-Xiebai-  Banxia decoction) | 9.98% |  | Shen Tong Zhu Yu Tang  (STZYT) | 5,934 |
| 5 | Jhih-Gan-Cao-Tang | 10.10% | Chuan-Cyong-Cha-Tiao-San | 9.24% |  | Shu-jing-huo-xue-tang | 10,363 |  | Tian-Wang-Bu-Xin-Dan | 9.54% |  | Ge Xia Zhu Yu Tang (GXZYT) | 5,273 |
| 6 | Chuan-Cyong-Cha-Tiao-San | 9.90% | Du-Huo-Ji-Sheng-Tang | 7.67% |  | Liu-wei-di-huang-wan | 9,709 |  | Yang-Xin-Tang | 6.66% |  | Tong Qiao Huo Xue Tang  (TQHXT) | 4,735 |
| 7 | Sie-Fu-Jhu-Yu-Tang | 9.00% | Sie-Fu-Jhu-Yu-Tang | 6.86% |  | Ji-sheng-shen-qui-wan | 9,210 |  | Zhen-Wu-Tang | 5.78% |  | Shu Jing Hwo Shiee Tang  (SJHST) | 4,518 |
| 8 | Ban-Sia-Sie-Sin-Tang | 8.50% | Yin-Ciao-San | 6.58% |  | Huang-qi-gui-zhi-wu-wu-tang | 8,394 |  | Jia-Wei-Xiao-Yao-San | 5.34% |  | Tao He Cheng Qi Tang  (THCQT) | 4,494 |
| 9 | Yin-Ciao-San | 8.40% | Ban-Sia-Sie-Sin-Tang | 6.55% |  | Zhi-gan0cao-tang | 6,898 |  | Fufang-Danshen-Pian | 4.56% |  | Bu Yang HuanWu Tang  (BYHWT) | 2,751 |
| 10 | Ma-Sing-Gan-Shih-Tang | 8.00% | Liou-Wei-Di-Huang-Wan | 6.41% |  | Xiao-xu-ming-tang | 5,971 |  | Ji-Sheng-Shen-Qi-Wan | 4.03% |  | Fu Fang Dan Shen Pian  (FFDSP) | 2,563 |

CeVD= Cerebrovascular disease; CVD= Cardiovascular disease; VTE= Venous thromboembolism; CM=Chinese medication (in terms of extract granules of Chinese medications); TCM=Traditional Chinese medicine; IHD= Ischemic heart disease; AF= Atrial fibrillation; CHM= Chinese herbal medication.

# The median interval between stroke onset to the first TCM consultation (including Chinese medications or acupuncture/traumatology treatment) was 12.2 months.

**
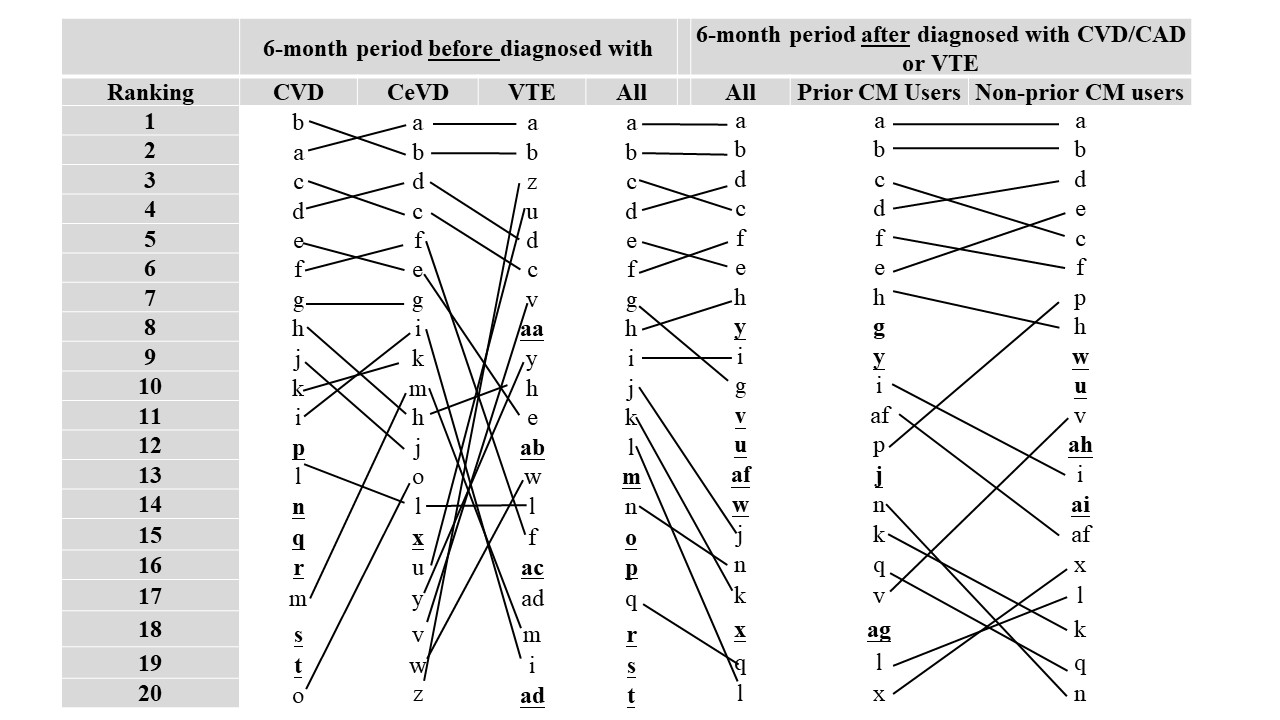
**

**Supplement Figure 2. Ranking of the top 20 most frequently prescribed single Chinese medicinal during 6-month periods before or after being diagnosed with new-onset cerebrovascular- or cardiovascular-related diseases**

Ranking: 1= most frequently prescribed Chinese medicinal formulas; 20= the 20^th^ frequent Chinese medicinal formulas; CAD= Coronary artery disease; CeVD= Cerebrovascular Disease; CVD= Cardiovascular disease; VTE= Venous thromboembolism; CM= Chinese medication (in terms of extract granules of Chinese medications); TCM= Traditional Chinese medicine; a= Yan Hu Suo (YHS); b= Dan Shen (DS); c= Jie Geng (JG); d=Ge Gen (GG); e= Huang Cin (HC); f= Chuan Bei Mu (CBM); g= Bai Jhih (BJ); h= Gan Cao (GC); i= Du Jhong (DJ); j= Ku Sing Ren (KSR); k= Mai Men Dong (MMD); l= Huang Ci (HC); m= Chuan Cyong (CC); n= Hou Pu (HP); o= Syuan Shen (SS); p= Siang Fu (SF); q= Yuh Jin YJ; r= Yu Sing Cao (YSC); s= Suan Zao Ren (SZR); t= Jhih Ke (JK); u= Syu Duan (SD); v= Chuan Niou Si (CNS); w= Ji Sie Teng (JST); x= Ye Jiao Teng (YJT); y= Da Huang (DH); z= Mu Gua (MG); aa= Hwang Bor (HB); ab= Yi Yi Ren (YYR); ac= Che Cian Zih (CCZ); ad= Bai Jhu (BJ); ae= Fu Ling (FL); af= San Ci (SC); ag= Hai Piao Siao (HPS); ah= Mei Yao (MY); aj= Ru Siang (RS)

Those highlighted single Chinese medicinal were unique specifically for either those patients newly diagnosed with CVD, CeVD, VTE or all patients before and/or after the diagnoses, as compared to the counterparts (e.g., Siang Fu (SF, as p), Hou Pu (HP, as n), Yuh Jin (YJ, as q), Yu Sing Cao (YSC, as r), Suan Zao Ren (SZR, as s), Jhih Ke (JK, as t) for CVD, as compared with CeVD and/or VTE during 6-month periods before being diagnosed.

**Reference**

Chang, C. C., Y. C. Lee, C. C. Lin, C. H. Chang, C. D. Chiu, L. W. Chou, M. F. Sun and H. R. Yen (2016). "Characteristics of traditional Chinese medicine usage in patients with stroke in Taiwan: A nationwide population-based study." J Ethnopharmacol **186**: 311-321.

Wang, L. S., P. T. Yen, S. F. Weng, J. H. Hsu and J. L. Yeh (2022). "Clinical Patterns of Traditional Chinese Medicine for Ischemic Heart Disease Treatment: A Population-Based Cohort Study." Medicina (Kaunas) **58**(7).

Zheng, L. C., H. Livneh, W. J. Chen, M. C. Lin, M. C. Lu, C. C. Yeh and T. Y. Tsai (2020). "Reduced Stroke Risk among Patients with Atrial Fibrillation Receiving Chinese Herbal Medicines Treatment: Analysis of Domestic Data in Taiwan." Medicina (Kaunas) **56**(6).
